# Supplementary material for: Temporal specificity of abnormal neural oscillations during phonatory events in laryngeal dystonia
Source: Brain Commun. 2022 Feb 11;4(2):fcac031. doi: 10.1093/braincomms/fcac031 (PMC8962453; doi:10.1093/braincomms/fcac031)
Supplement: fcac031_Supplementary_Data [file fcac031_supplementary_data.zip › Supplementary Material.pdf]

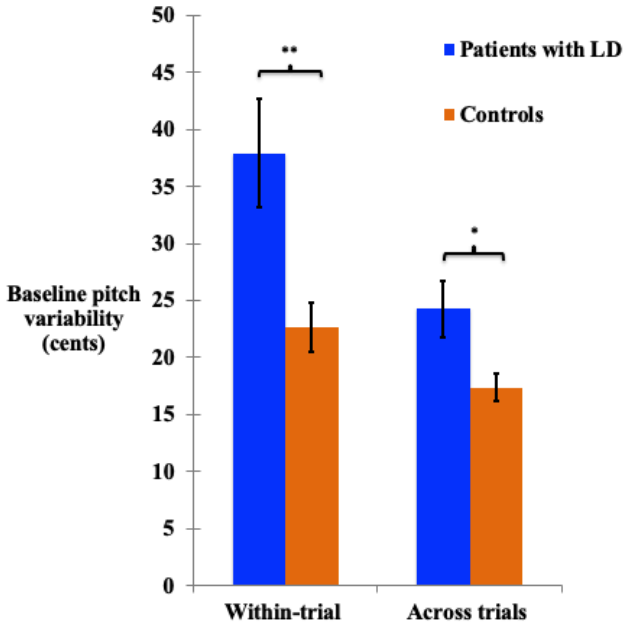

Supplementary Figure 1: Baseline pitch variability in patients with LD and controls

Baseline vocal range: Baseline pitch variability (200ms prior to perturbation onset) in patients with LD ($n = 15$) differs from that in controls ($n = 12$) both within-trial (two-sample heteroscedastic t-test, $t = 2.94$, $p = 0.0038$) and across trials (two-sample heteroscedastic t-test, $t = 2.5$, $p = 0.0202$) . Error bars indicate standard error of the mean variability.

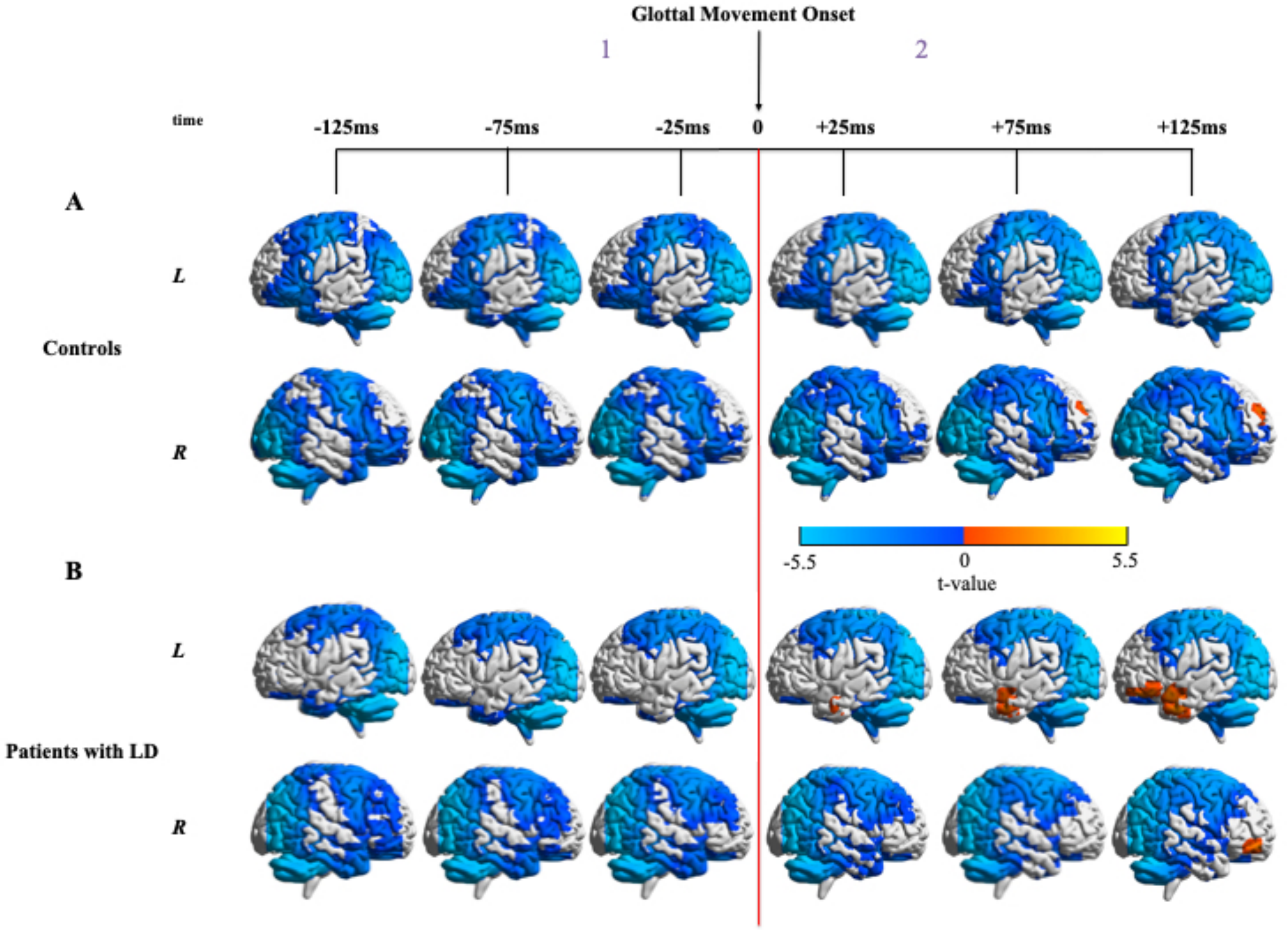

Supplementary Figure 2: Neural activity in controls and patients with LD in the beta band (12 - 30 Hz) locked to glottal movement onset

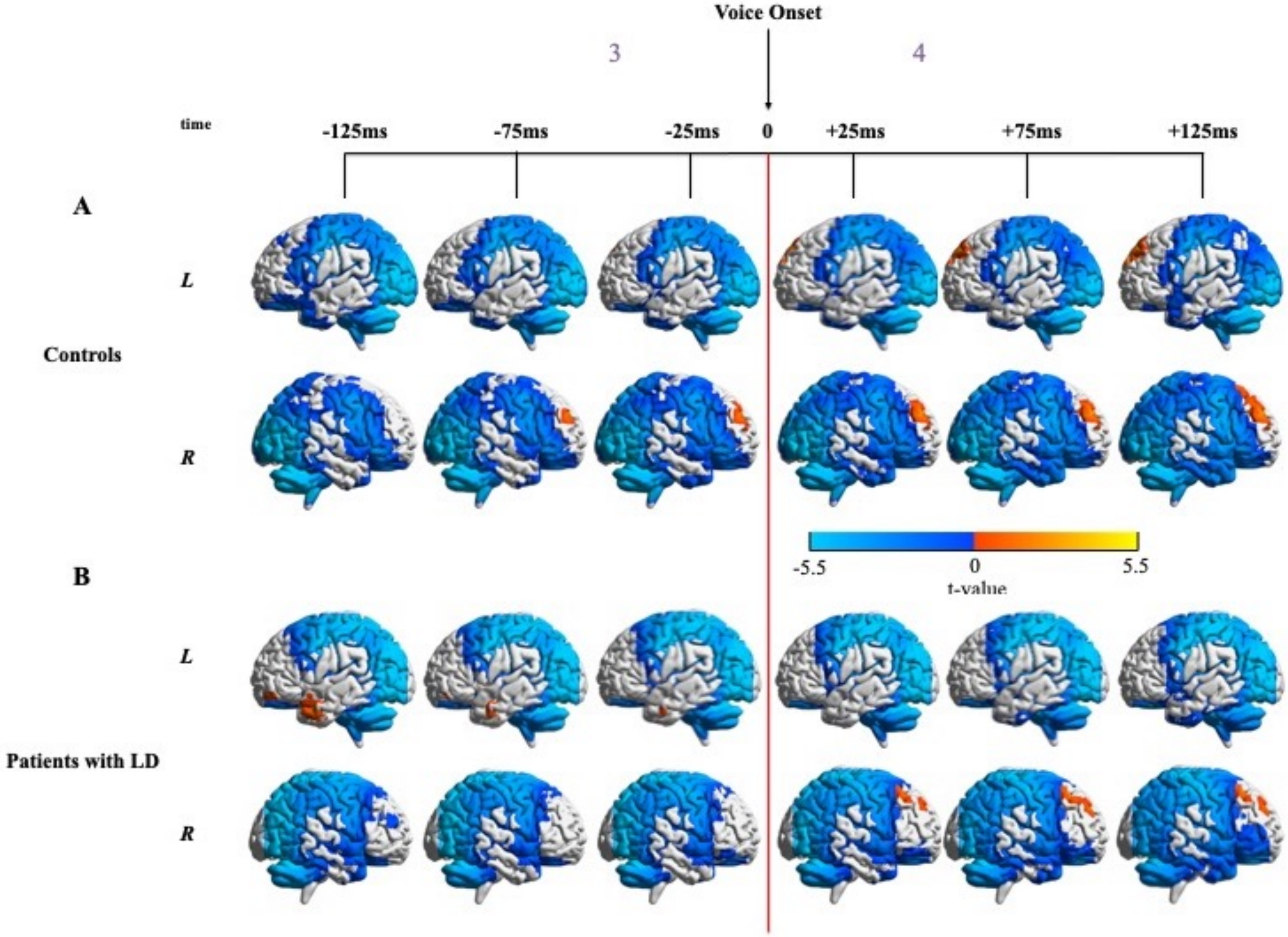

Supplementary Figure 3: Neural activity in controls and patients with LD in the beta band (12 - 30 Hz) locked to voice onset

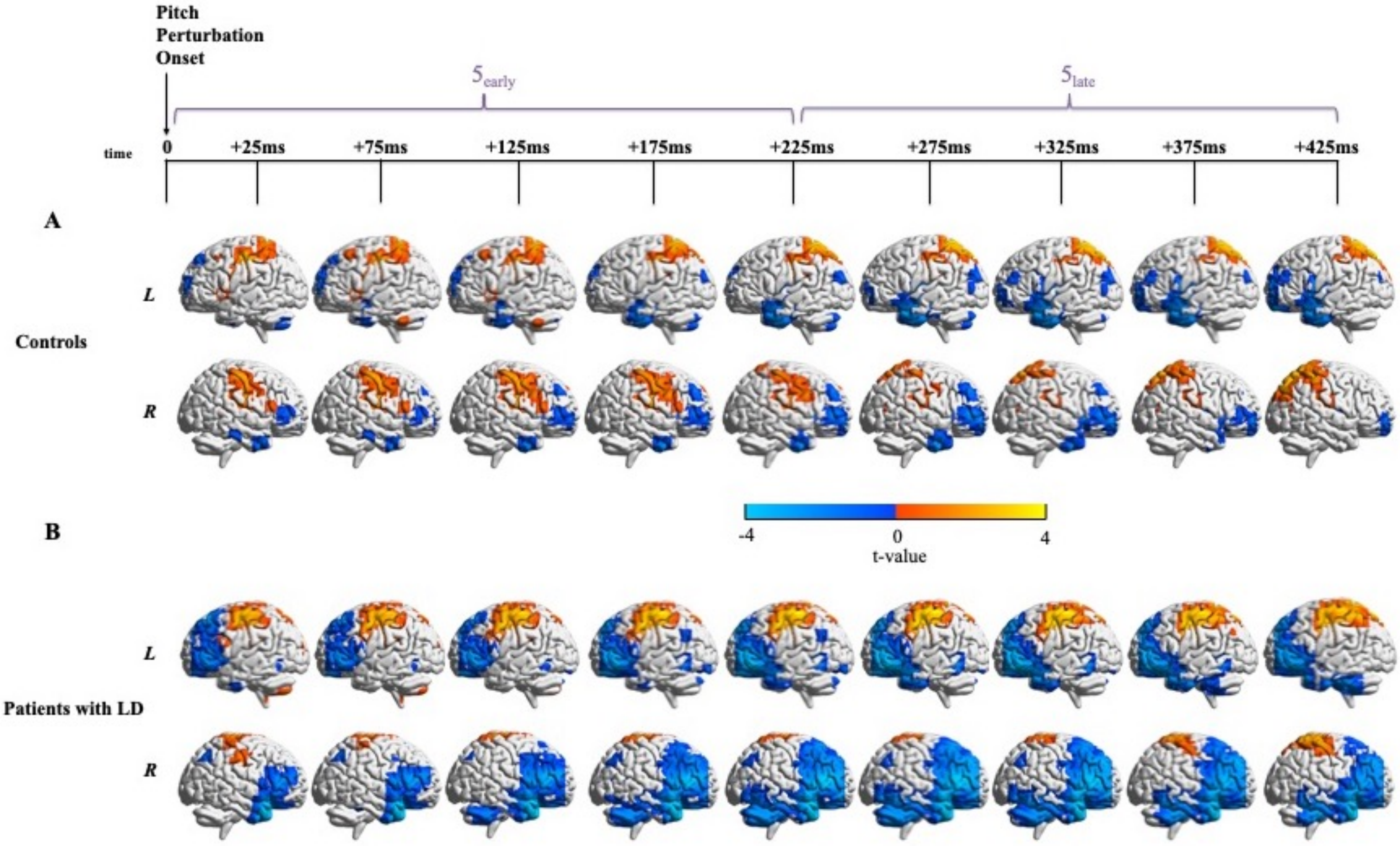

Supplementary Figure 4: Neural activity in controls and patients with LD in the beta band (12 - 30 Hz) locked to pitch perturbation onset

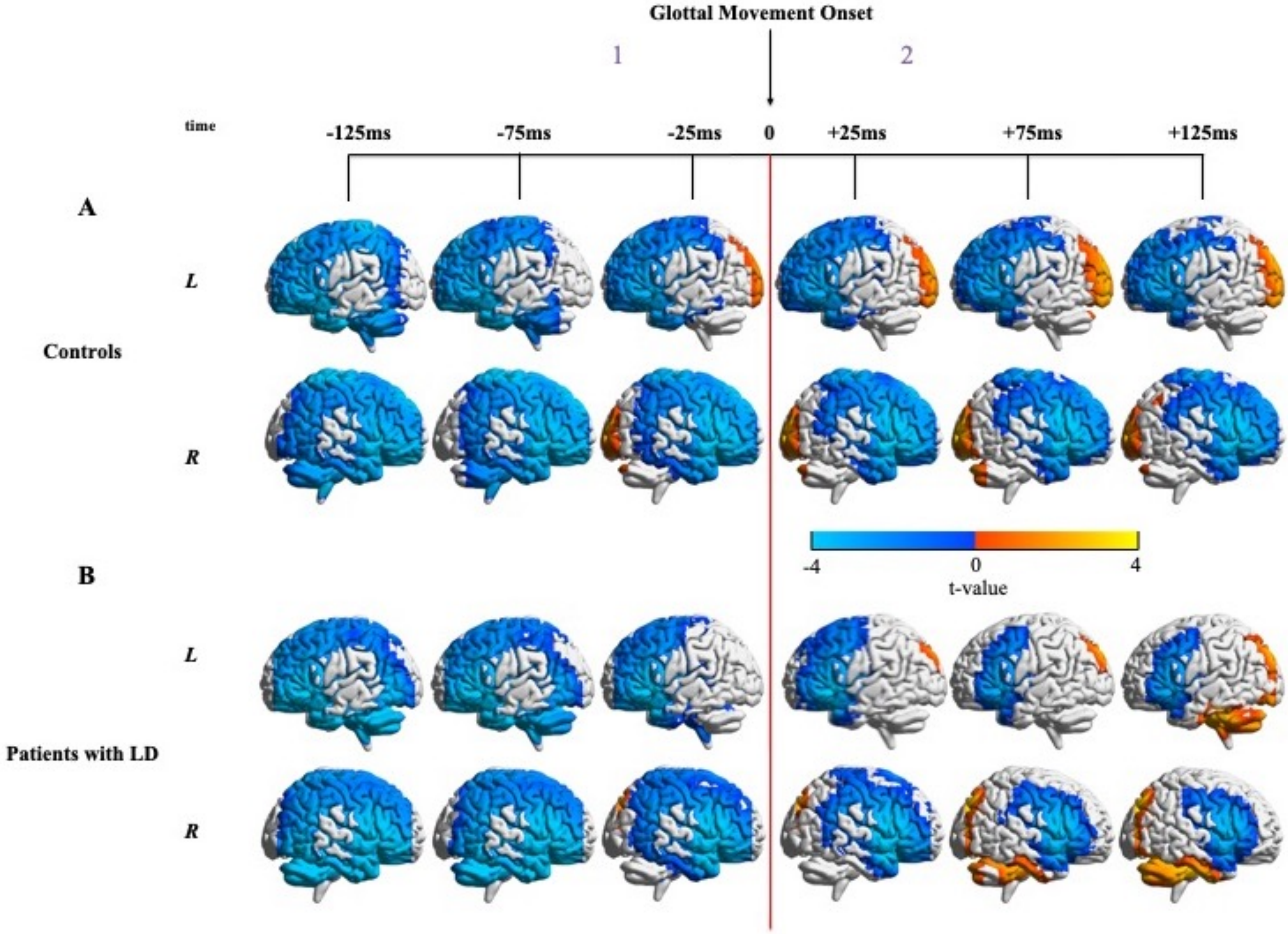

Supplementary Figure 5: Neural activity in controls and patients with LD in the high gamma band (65-150 Hz) locked to glottal movement onset

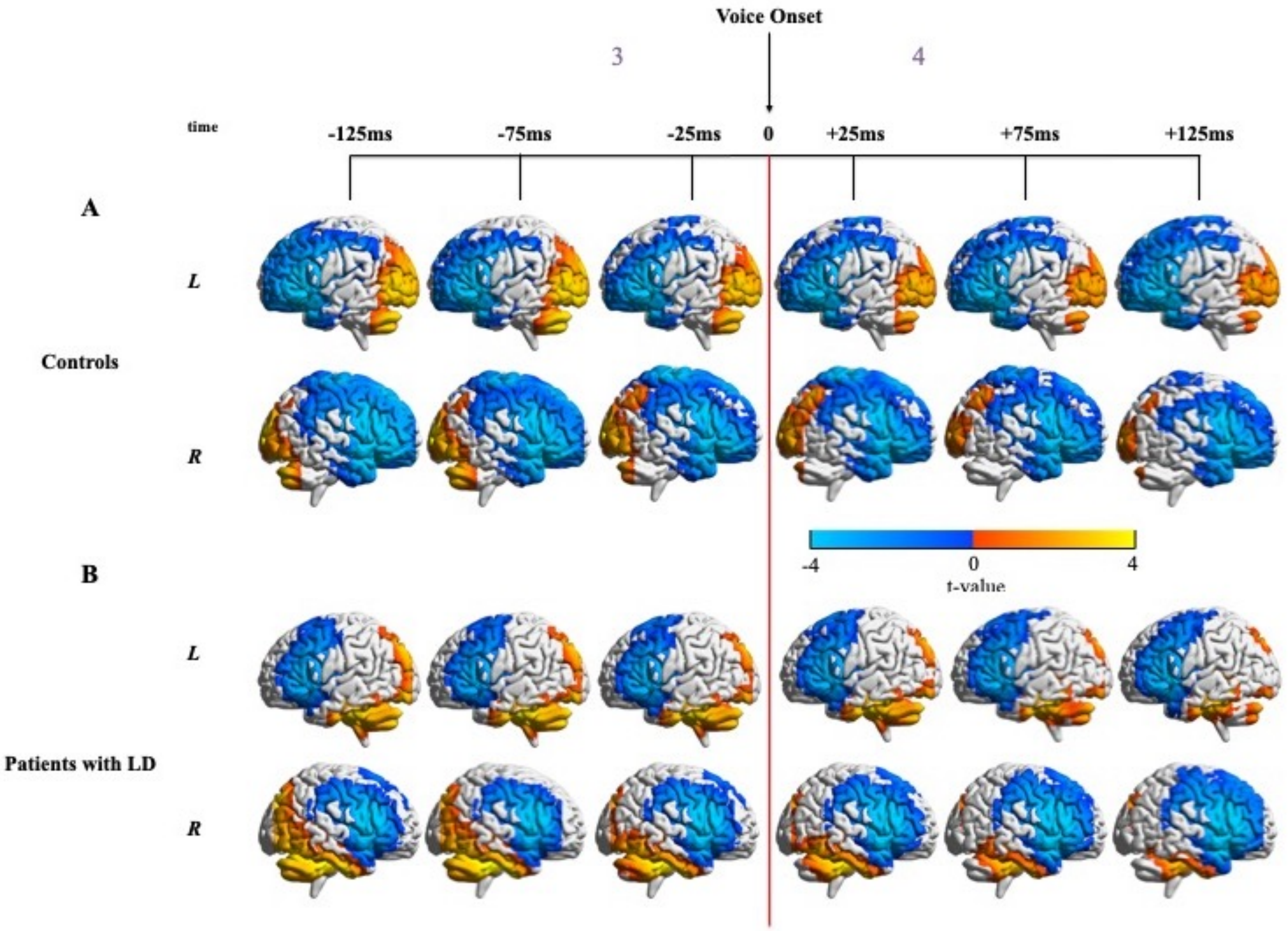

Supplementary Figure 6: Neural activity in controls and patients with LD in the high gamma band (65-150 Hz) locked to voice onset

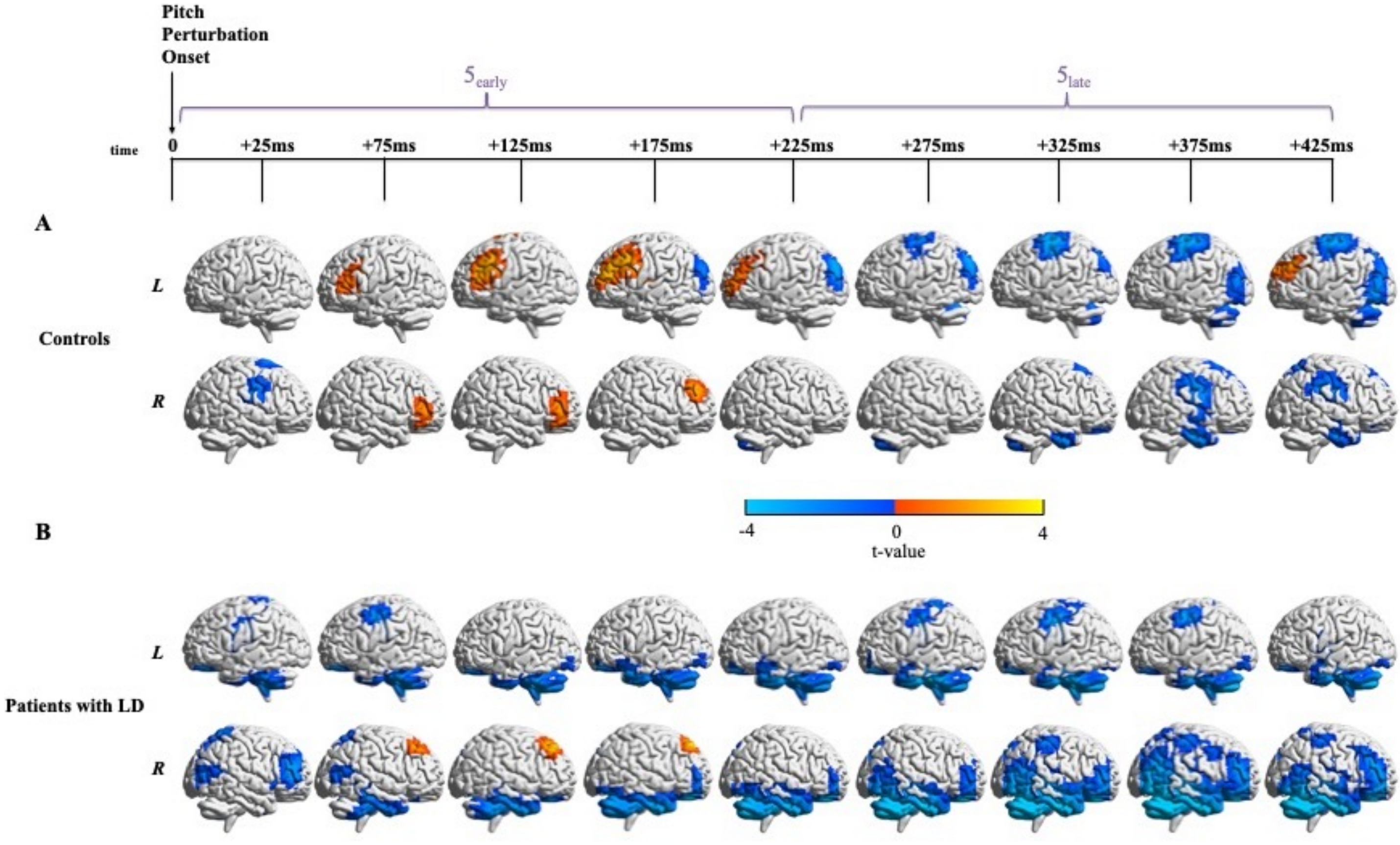

Supplementary Figure 7: Neural activity in controls and patients with LD in the high gamma band (65-150 Hz) locked to pitch perturbation onset

Supplementary Table 1: Meta-analysis of studies of the CNS in patients with Adductor LD

Study	Modality	Task	Regions with increase in activity	Regions with decrease in activity	Regions with increase in connectivity (Seed - Target)	Regions with decrease in connectivity (Seed - Target)
Haslinger B, et al. (2005), Neurology; 65(10): 1562-9.	Silent event-related fMRI	Prolonged vowel phonation		L superior M1 and S1, R inferior M1 and S1, ACC, mesial and L SMA, L and R dPMC, L SFG, R IFG, L MFG, R superior parietal,R occipital, L fusiform gyrus, L parahippocampal gyrus, L hemisphere of the cerebellum		
		Whispered speech		L superior M1, R inferior postcentral, L and R inferior parietal, mesial frontal, R STG, R MTG, R fusiform gyrus, R parieto-occipital		
Ali et al. (2006), Journal of Speech, Language, and Hearing Research; Vol. 49 1127-1146	$H_2{}^{15}O$ PET	Narrative speech	Cerebellum, dorsal and ventral precentral gyrus, anterior insula, ACC, A1, SII, posterior auditory association cortex	Posterior SMG, posterior MTG, dorsal postcentral gyrus, anterior auditory association cortex, PAG, SMA, anterior MTG		
Simonyan & Ludlow (2010), Cerebral Cortex; 20:2749--2759	fMRI	Symptomatic syllable production	M1, S1, Insula, STG, MTG, Cerebellum, Operculum, Basal Ganglia, Thalamus,	Midbrain		
		Asymptomatic whimper	M1, S1, Operculum, Insula, MCC, MTG	Insula, Thalamus, Basal Ganglia, Cerebellum, SMA		
		Coughing	M1, S1, Operculum, anterior insula, STG, MCC, MTG, Midbrain	Cerebellum		
		Voluntary breathing	M1, S1	Insula, Midbrain, Cerebellum, SMG		
Kiyuna, A., et al. (2017), J Voice; 31(3): p. 379 e1-379 e11.	fMRI	Reading five-digit numbers	L MTG, L thalamus, L and R precentral gyrus, L and R postcentral gyrus, R insula, R Cerebellum VIII and IX, R putamen, L Cerebellum I-IV, R SMA	L Cerebellum Crus 1 and 2, R Cerebellum Crus 1, L STG, L Cerebellum VI		
		Resting state			L Thalamus - L Caudate; R precentral gyrus - L ITG, L temporal pole, L MTG; L postcentral gyrus - R frontal pole; L inferior operculum - R precentral and postcentral gyri; Cerebellum (vermis I, II) - R lateral occipital cortex, R superior parietal lobule; R Cerebellum (IX) - R precentral and postcentral gyri	L insula - R angular gyrus, R lateral occipital cortex; R thalamus - L MFG, L IFG, pars triangularis; L precuneus - L and R lingual gyrus; R precentral gyrus - R occipital pole
Khosravani S, et al. (2019), Clinical Neurophysiology; 130(6): 1033-40.	EEG	Vowel vocalisation	L somatosensory-premotor cortices (late vocalisation, gamma band)	L motor cortex (early vocalisation, alpha band)		
Daliri A, et al. (2020), Journal of Speech, Language, and Hearing Research; 63(2): 421-32.	fMRI	Normal sentence production and Sentence production under masking noise	L ventral sensorimotor cortex, L anterior planum temporale, L posterior STG / planum temporale			
		Resting state			L mid-Rolandic cortex - L Heschl's gyrus, L posterior STG, R Heschl's gyrus; L ventral Rolandic cortex - L posterior STG; R mid-Rolandic cortex - L posterior STG	

Abbreviations used: fMRI = Functional Magnetic Resonance Imaging, PET = Positron Emission Tomography, EEG = Electroencephalography, L = left, R = right, M1 = primary motor cortex, S1 = primary somatosensory cortex, ACC = anterior cingulate cortex, SMA = supplementary motor area, dPMC = dorsal premotor cortex, SFG = superior frontal gyrus, IFG = inferior frontal gyrus, MFG = middle frontal gyrus, STG = superior temporal gyrus, MTG = middle temporal gyrus, A1 = primary auditory cortex, SII = secondary somatosensory cortex, SMG = supramarginal gyrus, PAG = periaqueductal grey, MCC = middle cingulate cortex, ITG = inferior temporal gyrus.

Supplementary Table 2: Peak voxels with significant beta-band activity differences with respect to glottal movement onset

Hemisphere	Peak	MNI Coordinates	Anatomical Labels	Time with respect to glottal movement onset
Left	1	[-41.2 -78.1 32.2]	Angular Gyrus (Brodmann Area 39)	-125 to +125ms
	2	[-40.4 -81.9 28.3]	Superior Occipital Gyrus (Brodmann Area 19)	-125 to +125ms
	3	[-41.9 -70.3 25.9]	Middle Temporal Gyrus (Brodmann Area 39)	-125ms
	4	[-23.3 -35.8 -25.7]	Cerebellar Anterior Lobe	-125ms
	5	[-59.0 -3.8 22.0]	Precentral Gyrus (Brodmann Area 4)	-125 to +25ms
	6	[-59.0 2.5 22.0]	Precentral Gyrus (Brodmann Area 6)	-125 to +75ms
	7	[-63.7 -29.0 27.5]	Inferior Parietal Lobule (Brodmann Area 40)	-125 to -25ms
	8	[-55.2 19.6 -18.4]	Superior Temporal Gyrus (Brodmann Area 38)	-125 to +125ms
	9	[-55.2 28.1 -8.9]	Inferior Frontal Gyrus (Brodmann Area 47)	-125 to +125ms
	10	[-55.9 4.1 20.4]	Inferior Frontal Gyrus (Brodmann Area 6)	-125 to +75ms
	11	[-48.2 -76.4 26.7]	Middle Temporal Gyrus (Brodmann Area 39)	-75 to +125ms
	12	[-58.3 3.3 15.6]	Inferior Frontal Gyrus (Brodmann Area 44)	+75 to +125ms
	13	[-51.3 21.9 8.5]	Inferior Frontal Gyrus (Brodmann Area 45)	+75ms
	14	[-47.4 43.5 -17.6]	Inferior Frontal Gyrus (Brodmann Area 47)	+125ms
	15	[-14.8 -48.6 -57.2]	Cerebellar Tonsil	+125ms
	16	[-59.0 5.7 12.5]	Precentral Gyrus (Brodmann Area 44)	+125ms
	17	[-52.0 12.0 -24.7]	Superior Temporal Gyrus (Brodmann Area 38)	+125ms
	18	[-52.0 9.5 -30.2]	Middle Temporal Gyrus (Brodmann Area 38)	+125ms
	19	[-30.3 17.3 60.7]	Middle Frontal Gyrus (Brodmann Area 6)	+125ms
Right	1	[41.9 -69.1 34.6]	Precuneus (Brodmann Area 39)	-125 to +25ms
	2	[41.9 -69.1 39.3]	Inferior Parietal Lobule (Brodmann Area 39)	-125 to +125ms
	3	[49.0 -72.2 37.0]	Angular Gyrus (Brodmann Area 39)	-125 to +25ms
	4	[60.1 -27.9 -27.1]	Inferior Temporal Gyrus (Brodmann Area 20)	-125ms
	5	[24.5 25.2 62.3]	Superior Frontal Gyrus (Brodmann Area 8)	-25 to +125ms
	6	[16.6 -77.8 34.6]	Precuneus (Brodmann Area 19)	+75ms
	7	[8.0 -83.0 47.0]	Precuneus (Brodmann Area 7)	+75 to +125ms
	8	[59.3 23.6 -1.0]	Inferior Frontal Gyrus (Brodmann Area 45)	+75 to +125ms
	9	[48.2 52.1 -8.9]	Middle Frontal Gyrus (Brodmann Area 10)	+75 to +125ms
	10	[31.6 -84.1 -14.4]	Middle Occipital Gyrus (Brodmann Area 19)	+75 to +125ms
	11	[23.7 -77.0 -24.7]	Cerebellar Posterior Lobe	+75 to +125ms
	12	[22.1 -75.4 -9.7]	Lingual Gyrus	+75 to +125ms
	13	[49.8 -58.0 -16.8]	Fusiform Gyrus	+125ms
	14	[39.5 -53.2 44.9]	Inferior Parietal Lobule	+125ms

Supplementary Table 3: Peak voxels with significant high-gamma-band activity differences with respect to glottal movement onset

Hemisphere	Peak	MNI Coordinates	Anatomical Labels	Time with respect to glottal movement onset
Left	1	[-64.0 -27.0 39.0]	Postcentral Gyrus (Brodmann Area 40)	+25 to +125ms
	2	[-65.3 -25.5 -8.4]	Middle Temporal Gyrus (Brodmann Area 21)	+125ms
	3	[-61.4 5.6 -2.9]	Superior Temporal Gyrus (Brodmann Area 22)	+125ms
Right	1	[46.6 -74.2 -57]	Cerebellar Posterior Lobe	-125 to -75ms
	2	[48.0 37.0 -9.0]	Inferior Frontal Gyrus (Brodmann Area 47)	+75 to +125ms
	3	[46.6 4.4 -40.6]	Middle Temporal Gyrus (Brodmann Area 21)	+75 to +125ms
	4	[62.1 -26.9 8.2]	Superior Temporal Gyrus (Brodmann Area 41)	+125ms

Supplementary Table 4: Peak voxels with significant beta-band activity differences with respect to voice onset

Hemisphere	Peak	MNI Coordinates	Anatomical Labels	Time with respect to voice onset
Left	1	[-54.4 14.2 -27.1]	Superior Temporal Gyrus (Brodmann Area 38)	-125 to -25ms
	2	[-54.4 9.5 -32.6]	Middle Temporal Gyrus (Brodmann Area 38)	-125 to -75ms
	3	[-33.4 42.0 -17.6]	Middle Frontal Gyrus (Brodmann Area 47)	-125ms
	4	[-55.2 20.4 14.0]	Inferior Frontal Gyrus (Brodmann Area 45)	-125 to -75ms
	5	[-31.8 11.1 60.7]	Middle Frontal Gyrus (Brodmann Area 6)	-125 to +25ms
	6	[-14.8 -13.7 68.6]	Superior Frontal Gyrus (Brodmann Area 6)	-125 to -75ms
	7	[-38.8 -27.5 45.7]	Postcentral Gyrus (Brodmann Area 2)	-125 to -75ms
	8	[-39.6 -76.5 38.6]	Precuneus (Brodmann Area 19)	-125 to -75ms
	9	[-39.6 -83.5 27.5]	Superior Occipital Gyrus (Brodmann Area 19)	-125 to -75ms
	10	[-42.7 -79.5 30.6]	Angular Gyrus (Brodmann Area 39)	-125 to -75ms
	11	[-31.8 -27.5 69.4]	Precentral Gyrus (Brodmann Area 4)	-25 to +125ms
	12	[-52.8 -26.7 58.3]	Postcentral Gyrus (Brodmann Area 1)	-25 to +125ms
	13	[-31.1 -85.0 -11.2]	Inferior Occipital Gyrus (Brodmann Area 18)	-25 to +125ms
	14	[-15.5 -58.7 -57.9]	Cerebellar Tonsil	-25 to +125ms
Right	1	[22.9 -66.7 62.3]	Superior Parietal Lobule (Brodmann Area 7)	-125 to -75ms
	2	[22.1 -62.0 61.5]	Superior Parietal Lobule (Brodmann Area 7)	-125 to -75ms
	3	[34.0 -35.8 69.4]	Postcentral Gyrus (Brodmann Area 1)	-125 to +125ms
	4	[25.3 -84.9 -16.8]	Middle Occipital Gyrus (Brodmann Area 19)	-125 to +125ms
	5	[56.9 -59.6 -17.6]	Inferior Temporal Gyrus (Brodmann Area 37)	-25 to +125ms
	6	[47.4 -50.9 -50.0]	Cerebellar Tonsil	-25 to +125ms
	7	[34.0 -28.7 67.0]	Precentral Gyrus (Brodmann Area 4)	+25 to +125ms
	8	[6.3 -3.3 67.0]	Superior Frontal Gyrus (Brodmann Area 6)	+25 to +75ms
	9	[53.7 20.4 13.3]	Inferior Frontal Gyrus (Brodmann Area 44)	+75 to +125ms
	10	[41.1 22.0 37.0]	Middle Frontal Gyrus (Brodmann Area 8)	+75 to +125ms
	11	[6.3 3.8 69.4]	Superior Frontal Gyrus (Brodmann Area 6)	+125ms

Supplementary Table 5: Peak voxels with significant high-gamma-band activity differences with respect to voice onset

Hemisphere	Peak	MNI Coordinates	Anatomical Labels	Time with respect to voice onset
Left	1	[-8.0 53.0 39.0]	Superior Frontal Gyrus (Brodmann Area 9)	-125ms
	2	[-64.0 -19.0 23.0]	Postcentral Gyrus (Brodmann Area 1)	-125 to -25ms
	3	[-16.0 61.0 -17.0]	Superior Frontal Gyrus (Brodmann Area 11)	-125 to +125ms
	4	[-56.0 -11.0 -17.0]	Middle Temporal Gyrus (Brodmann Area 21)	-125 to +125ms
Right	1	[41.3 6.5 -42.2]	Inferior Temporal Gyrus (Brodmann Area 20)	-125 to +25ms
	2	[48.0 -19.0 -33.0]	Inferior Temporal Gyrus (Brodmann Area 20)	-125 to +25ms
	3	[64.0 -27.5 8.2]	Superior Temporal Gyrus (Brodmann Area 41)	-125 to +125ms
	4	[8.0 -67.0 39.0]	Precuneus (Brodmann Area 7)	-125 to -75ms
	5	[46.8 -68.5 45.2]	Inferior Parietal Lobule (Brodmann Area 39)	+75ms

Supplementary Table 6: Peak voxels with significant beta-band activity differences with respect to pitch perturbation onset

Hemisphere	Peak	MNI Coordinates	Anatomical Labels	Time with respect to voice onset
Left	1	[-39.6 -75.0 -50.8]	Cerebellar Inferior Semi-Lunar Lobule	+25 to +225ms
	2	[-48.2 20.4 4.6]	Inferior Frontal Gyrus (Brodmann Area 45)	+25 to +175ms
	3	[-38.8 23.5 44.1]	Middle Frontal Gyrus (Brodmann Area 8)	+25ms
	4	[-25.6 -59.0 44.9]	Superior Parietal Lobule (Brodmann Area 7)	+175 to +375ms
	5	[-52.8 27.0 -11.2]	Inferior Frontal Gyrus (Brodmann Area 47)	+225ms
	6	[-25.6 -51.9 43.3]	Precuneus (Brodmann Area 7)	+225 to +375ms
	7	[-46.6 34.9 -11.2]	Inferior Frontal Gyrus (Brodmann Area 47)	+275ms
Right	1	[48.0 21.0 -25.0]	Superior Temporal Gyrus (Brodmann Area 38)	+25 to +425ms
	2	[62.4 26.0 10.1]	Inferior Frontal Gyrus (Brodmann Area 9)	+25 to +425ms
	3	[54.5 20.4 14.8]	Inferior Frontal Gyrus (Brodmann Area 44)	+25 to +325ms
	4	[49.8 -3.3 33.8]	Precentral Gyrus (Brodmann Area 6)	+75 to +275ms
	5	[49.8 3.8 38.6]	Middle Frontal Gyrus (Brodmann Area 6)	+125 to +275ms
	6	[55.3 3.8 31.4]	Inferior Frontal Gyrus (Brodmann Area 6)	+125 to +275ms
	7	[55.3 26.0 14.0]	Inferior Frontal Gyrus (Brodmann Area 9)	+125 to +325ms
	8	[55.3 -67.5 -41.3]	Cerebellar Posterior Lobe	+175 to +325ms
	9	[18.2 52.9 38.6]	Superior Frontal Gyrus (Brodmann Area 9)	+225 to +325ms
	10	[15.0 62.4 7.7]	Superior Frontal Gyrus (Brodmann Area 10)	+375 to +425ms
	11	[55.3 -52.5 -43.7]	Cerebellar Tonsil	+375 to +425ms
	12	[45.8 -66.7 45.7]	Inferior Parietal Lobule (Brodmann Area 39)	+375 to +425ms
	13	[63.2 -12.1 -16.0]	Middle Temporal Gyrus (Brodmann Area 21)	+425ms

Supplementary Table 7: Peak voxels with significant high-gamma-band activity differences with respect to pitch perturbation onset

Hemisphere	Peak	MNI Coordinates	Anatomical Labels	Time with respect to pitch perturbation onset
Left	1	[-32.0 29.0 7.0]	Inferior Frontal Gyrus (Brodmann Area 45)	+25 to +125ms
	2	[10/9 35.1 -26.5]	Rectal Gyrus (Brodmann Area 11)	+25 to +425ms
	3	[-38.1 -42.0 -54.0]	Cerebellar Tonsil	+25 to +425ms
	4	[-31.1 -81.9 38.1]	Angular Gyrus (Brodmann Area 39)	+175 to +275ms
	5	[-48.0 -20.7 -34.3]	Inferior Temporal Gyrus (Brodmann Area 20)	+225 to +325ms
Right	1	[52.3 37.2 17.6]	Middle Frontal Gyrus (Brodmann Area 46)	+25 to +225ms
	2	[14.6 29.3 -25.0]	Orbital Gyrus (Brodmann Area 47)	+125 to +425ms
	3	[56.0 -27.5 -24.9]	Inferior Temporal Gyrus (Brodmann Area 20)	+125 to +425ms
	4	[48.0 45.0 23.0]	Middle Frontal Gyrus (Brodmann Area 46)	+125 to +225ms
	5	[56.3 -74.0 -33.0]	Cerebellar Posterior Lobe	+175 to +425ms
	6	[8.0 -91.0 31.0]	Cuneus (Brodmann Area 19)	+175 to +275ms
	7	[46.0 -84.3 -16.2]	Inferior Occipital Gyrus (Brodmann Area 18)	+225 to +425ms
	8	[24.0 33.3 54.6]	Superior Frontal Gyrus (Brodmann Area 8)	+325ms
