# Supplementary material for: Temporal specificity of abnormal neural oscillations during phonatory events in laryngeal dystonia
Source: Brain Commun. 2022 Feb 11;4(2):fcac031. doi: 10.1093/braincomms/fcac031 (PMC8962453; doi:10.1093/braincomms/fcac031)
Supplement: fcac031_Supplementary_Data [file fcac031_supplementary_data.zip › Supplementary Tables.docx]

**
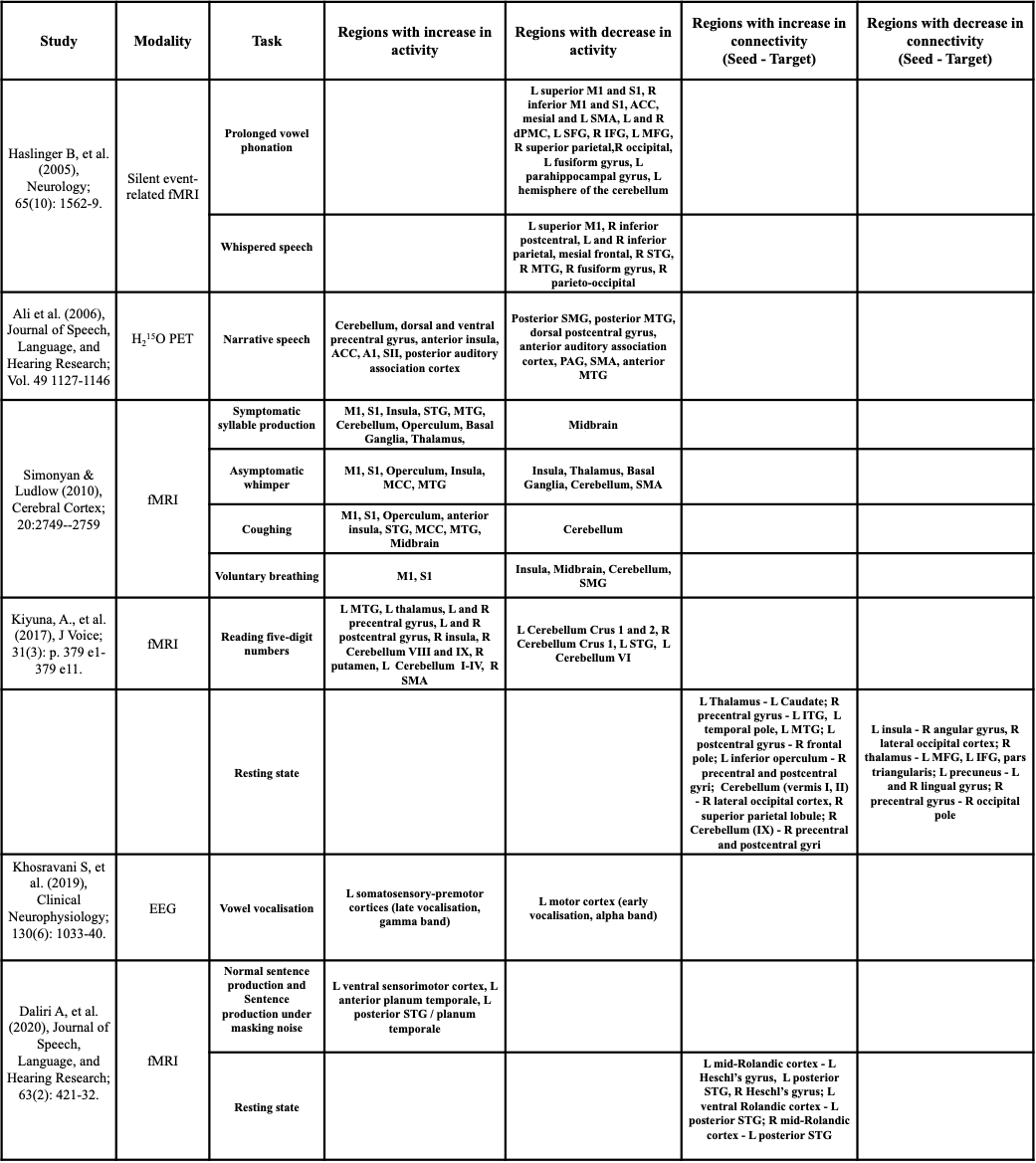
**

**Supplementary Table 1: Meta-analysis of studies of the CNS in patients with Adductor LD**

Abbreviations used: fMRI = Functional Magnetic Resonance Imaging, PET = Positron Emission Tomography, EEG = Electroencephalography, L = left, R = right, M1 = primary motor cortex, S1 = primary somatosensory cortex, ACC = anterior cingulate cortex, SMA = supplementary motor area, dPMC = dorsal premotor cortex, SFG = superior frontal gyrus, IFG = inferior frontal gyrus, MFG = middle frontal gyrus, STG = superior temporal gyrus, MTG = middle temporal gyrus, A1 = primary auditory cortex, SII = secondary somatosensory cortex, SMG = supramarginal gyrus, PAG = periaqueductal grey, MCC = middle cingulate cortex, ITG = inferior temporal gyrus.

**Supplementary Table 2: Number of participants in each analysis**

| **Analysis** | **Controls** | **Patients** |
| --- | --- | --- |
| Phonatory onset interval | 11 | 15 |
| Beta band and high gamma band MEG activity around glottal movement onset | 11 | 15 |
| Beta band and high gamma band MEG activity around voice onset | 11 | 15 |
| Behavioural response to pitch perturbation onset | 12 | 17 |
| Correlation between mean compensation to pitch perturbation and laryngeal diadochokinesis rate^a^ | 11 | 12 |
| Beta band MEG activity after pitch perturbation onset | 11 | 15 |
| High gamma band MEG activity after pitch perturbation onset^b^ | 16 | 15 |

^a^The number of participants is lower for this analysis because only those participants who underwent clinical voice evaluation and had good vocal response data could be included.

^b^Five controls were added to this analysis to improve signal-to-noise ratio.

| **Hemisphere**  **Supplementary Table 3: Peak voxels with significant beta-band activity differences with respect to glottal movement onset** | **Peak** | **MNI Coordinates** | **Anatomical Labels** | **Time with respect to glottal movement onset** |
| --- | --- | --- | --- | --- |
| Left | 1 | [-41.2 -78.1 32.2] | Angular Gyrus (Brodmann Area 39) | -125 to +125ms |
|  | 2 | [-40.4 -81.9 28.3] | Superior Occipital Gyrus (Brodmann Area 19) | -125 to +125ms |
|  | 3 | [-41.9 -70.3 25.9] | Middle Temporal Gyrus (Brodmann Area 39) | -125ms |
|  | 4 | [-23.3 -35.8 -25.7] | Cerebellar Anterior Lobe | -125ms |
|  | 5 | [-59.0 -3.8 22.0] | Precentral Gyrus (Brodmann Area 4) | -125 to +25ms |
|  | 6 | [-59.0 2.5 22.0] | Precentral Gyrus (Brodmann Area 6) | -125 to +75ms |
|  | 7 | [-63.7 -29.0 27.5] | Inferior Parietal Lobule (Brodmann Area 40) | -125 to -25ms |
|  | 8 | [-55.2 19.6 -18.4] | Superior Temporal Gyrus (Brodmann Area 38) | -125 to +125ms |
|  | 9 | [-55.2 28.1 -8.9] | Inferior Frontal Gyrus (Brodmann Area 47) | -125 to +125ms |
|  | 10 | [-55.9 4.1 20.4] | Inferior Frontal Gyrus (Brodmann Area 6) | -125 to +75ms |
|  | 11 | [-48.2 -76.4 26.7] | Middle Temporal Gyrus (Brodmann Area 39) | -75 to +125ms |
|  | 12 | [-58.3 3.3 15.6] | Inferior Frontal Gyrus (Brodmann Area 44) | +75 to +125ms |
|  | 13 | [-51.3 21.9 8.5] | Inferior Frontal Gyrus (Brodmann Area 45) | +75ms |
|  | 14 | [-47.4 43.5 -17.6] | Inferior Frontal Gyrus (Brodmann Area 47) | +125ms |
|  | 15 | [-14.8 -48.6 -57.2] | Cerebellar Tonsil | +125ms |
|  | 16 | [-59.0 5.7 12.5] | Precentral Gyrus (Brodmann Area 44) | +125ms |
|  | 17 | [-52.0 12.0 -24.7] | Superior Temporal Gyrus (Brodmann Area 38) | +125ms |
|  | 18 | [-52.0 9.5 -30.2] | Middle Temporal Gyrus (Brodmann Area 38) | +125ms |
|  | 19 | [-30.3 17.3 60.7] | Middle Frontal Gyrus (Brodmann Area 6) | +125ms |
| Right | 1 | [41.9 -69.1 34.6] | Precuneus (Brodmann Area 39) | -125 to +25ms |
|  | 2 | [41.9 -69.1 39.3] | Inferior Parietal Lobule (Brodmann Area 39) | -125 to +125ms |
|  | 3 | [49.0 -72.2 37.0] | Angular Gyrus (Brodmann Area 39) | -125 to +25ms |
|  | 4 | [60.1 -27.9 -27.1] | Inferior Temporal Gyrus (Brodmann Area 20) | -125ms |
|  | 5 | [24.5 25.2 62.3] | Superior Frontal Gyrus (Brodmann Area 8) | -25 to +125ms |
|  | 6 | [16.6 -77.8 34.6] | Precuneus (Brodmann Area 19) | +75ms |
|  | 7 | [8.0 -83.0 47.0] | Precuneus (Brodmann Area 7) | +75 to +125ms |
|  | 8 | [59.3 23.6 -1.0] | Inferior Frontal Gyrus (Brodmann Area 45) | +75 to +125ms |
|  | 9 | [48.2 52.1 -8.9] | Middle Frontal Gyrus (Brodmann Area 10) | +75 to +125ms |
|  | 10 | [31.6 -84.1 -14.4] | Middle Occipital Gyrus (Brodmann Area 19) | +75 to +125ms |
|  | 11 | [23.7 -77.0 -24.7] | Cerebellar Posterior Lobe | +75 to +125ms |
|  | 12 | [22.1 -75.4 -9.7] | Lingual Gyrus | +75 to +125ms |
|  | 13 | [49.8 -58.0 -16.8] | Fusiform Gyrus | +125ms |
|  | 14 | [39.5 -53.2 44.9] | Inferior Parietal Lobule | +125ms |

**Supplementary Table 4: Peak voxels with significant high-gamma-band activity differences with respect to glottal movement onset**

| **Hemisphere** | **Peak** | **MNI Coordinates** | **Anatomical Labels** | **Time with respect to glottal movement onset** |
| --- | --- | --- | --- | --- |
| Left | 1 | [-64.0 -27.0 39.0] | Postcentral Gyrus (Brodmann Area 40) | +25 to +125ms |
|  | 2 | [-65.3 -25.5 -8.4] | Middle Temporal Gyrus (Brodmann Area 21) | +125ms |
|  | 3 | [-61.4 5.6 -2.9] | Superior Temporal Gyrus (Brodmann Area 22) | +125ms |
| Right | 1 | [46.6 -74.2 -57] | Cerebellar Posterior Lobe | -125 to -75ms |
|  | 2 | [48.0 37.0 -9.0] | Inferior Frontal Gyrus (Brodmann Area 47) | +75 to +125ms |
|  | 3 | [46.6 4.4 -40.6] | Middle Temporal Gyrus (Brodmann Area 21) | +75 to +125ms |
|  | 4 | [62.1 -26.9 8.2] | Superior Temporal Gyrus (Brodmann Area 41) | +125ms |

**Supplementary Table 5: Peak voxels with significant beta-band activity differences with respect to voice onset**

| **Hemisphere** | **Peak** | **MNI Coordinates** | **Anatomical Labels** | **Time with respect to voice onset** |
| --- | --- | --- | --- | --- |
| Left | 1 | [-54.4 14.2 -27.1] | Superior Temporal Gyrus (Brodmann Area 38) | -125 to -25ms |
|  | 2 | [-54.4 9.5 -32.6] | Middle Temporal Gyrus (Brodmann Area 38) | -125 to -75ms |
|  | 3 | [-33.4 42.0 -17.6] | Middle Frontal Gyrus (Brodmann Area 47) | -125ms |
|  | 4 | [-55.2 20.4 14.0] | Inferior Frontal Gyrus (Brodmann Area 45) | -125 to -75ms |
|  | 5 | [-31.8 11.1 60.7] | Middle Frontal Gyrus (Brodmann Area 6) | -125 to +25ms |
|  | 6 | [-14.8 -13.7 68.6] | Superior Frontal Gyrus (Brodmann Area 6) | -125 to -75ms |
|  | 7 | [-38.8 -27.5 45.7] | Postcentral Gyrus (Brodmann Area 2) | -125 to -75ms |
|  | 8 | [-39.6 -76.5 38.6] | Precuneus (Brodmann Area 19) | -125 to -75ms |
|  | 9 | [-39.6 -83.5 27.5] | Superior Occipital Gyrus (Brodmann Area 19) | -125 to -75ms |
|  | 10 | [-42.7 -79.5 30.6] | Angular Gyrus (Brodmann Area 39) | -125 to -75ms |
|  | 11 | [-31.8 -27.5 69.4] | Precentral Gyrus (Brodmann Area 4) | -25 to +125ms |
|  | 12 | [-52.8 -26.7 58.3] | Postcentral Gyrus (Brodmann Area 1) | -25 to +125ms |
|  | 13 | [-31.1 -85.0 -11.2] | Inferior Occipital Gyrus (Brodmann Area 18) | -25 to +125ms |
|  | 14 | [-15.5 -58.7 -57.9] | Cerebellar Tonsil | -25 to +125ms |
| Right | 1 | [22.9 -66.7 62.3] | Superior Parietal Lobule (Brodmann Area 7) | -125 to -75ms |
|  | 2 | [22.1 -62.0 61.5] | Superior Parietal Lobule (Brodmann Area 7) | -125 to -75ms |
|  | 3 | [34.0 -35.8 69.4] | Postcentral Gyrus (Brodmann Area 1) | -125 to +125ms |
|  | 4 | [25.3 -84.9 -16.8] | Middle Occipital Gyrus (Brodmann Area 19) | -125 to +125ms |
|  | 5 | [56.9 -59.6 -17.6] | Inferior Temporal Gyrus (Brodmann Area 37) | -25 to +125ms |
|  | 6 | [47.4 -50.9 -50.0] | Cerebellar Tonsil | -25 to +125ms |
|  | 7 | [34.0 -28.7 67.0] | Precentral Gyrus (Brodmann Area 4) | +25 to +125ms |
|  | 8 | [6.3 -3.3 67.0] | Superior Frontal Gyrus (Brodmann Area 6) | +25 to +75ms |
|  | 9 | [53.7 20.4 13.3] | Inferior Frontal Gyrus (Brodmann Area 44) | +75 to +125ms |
|  | 10 | [41.1 22.0 37.0] | Middle Frontal Gyrus (Brodmann Area 8) | +75 to +125ms |
|  | 11 | [6.3 3.8 69.4] | Superior Frontal Gyrus (Brodmann Area 6) | +125ms |

**Supplementary Table 6: Peak voxels with significant high-gamma-band activity differences with respect to voice onset**

| **Hemisphere** | **Peak** | **MNI Coordinates** | **Anatomical Labels** | **Time with respect to voice onset** |
| --- | --- | --- | --- | --- |
| Left | 1 | [-8.0 53.0 39.0] | Superior Frontal Gyrus (Brodmann Area 9) | -125ms |
|  | 2 | [-64.0 -19.0 23.0] | Postcentral Gyrus (Brodmann Area 1) | -125 to -25ms |
|  | 3 | [-16.0 61.0 -17.0] | Superior Frontal Gyrus (Brodmann Area 11) | -125 to +125ms |
|  | 4 | [-56.0 -11.0 -17.0] | Middle Temporal Gyrus (Brodmann Area 21) | -125 to +125ms |
| Right | 1 | [41.3 6.5 -42.2] | Inferior Temporal Gyrus (Brodmann Area 20) | -125 to +25ms |
|  | 2 | [48.0 -19.0 -33.0] | Inferior Temporal Gyrus (Brodmann Area 20) | -125 to +25ms |
|  | 3 | [64.0 -27.5 8.2] | Superior Temporal Gyrus (Brodmann Area 41) | -125 to +125ms |
|  | 4 | [8.0 -67.0 39.0] | Precuneus (Brodmann Area 7) | -125 to -75ms |
|  | 5 | [46.8 -68.5 45.2] | Inferior Parietal Lobule (Brodmann Area 39) | +75ms |

**Supplementary Table 7: Peak voxels with significant beta-band activity differences with respect to pitch perturbation onset**

| **Hemisphere** | **Peak** | **MNI Coordinates** | **Anatomical Labels** | **Time with respect to voice onset** |
| --- | --- | --- | --- | --- |
| Left | 1 | [-39.6 -75.0 -50.8] | Cerebellar Inferior Semi-Lunar Lobule | +25 to +225ms |
|  | 2 | [-48.2 20.4 4.6] | Inferior Frontal Gyrus (Brodmann Area 45) | +25 to +175ms |
|  | 3 | [-38.8 23.5 44.1] | Middle Frontal Gyrus (Brodmann Area 8) | +25ms |
|  | 4 | [-25.6 -59.0 44.9] | Superior Parietal Lobule (Brodmann Area 7) | +175 to +375ms |
|  | 5 | [-52.8 27.0 -11.2] | Inferior Frontal Gyrus (Brodmann Area 47) | +225ms |
|  | 6 | [-25.6 -51.9 43.3] | Precuneus (Brodmann Area 7) | +225 to +375ms |
|  | 7 | [-46.6 34.9 -11.2] | Inferior Frontal Gyrus (Brodmann Area 47) | +275ms |
| Right | 1 | [48.0 21.0 -25.0] | Superior Temporal Gyrus (Brodmann Area 38) | +25 to +425ms |
|  | 2 | [62.4 26.0 10.1] | Inferior Frontal Gyrus (Brodmann Area 9) | +25 to +425ms |
|  | 3 | [54.5 20.4 14.8] | Inferior Frontal Gyrus (Brodmann Area 44) | +25 to +325ms |
|  | 4 | [49.8 -3.3 33.8] | Precentral Gyrus (Brodmann Area 6) | +75 to +275ms |
|  | 5 | [49.8 3.8 38.6] | Middle Frontal Gyrus (Brodmann Area 6) | +125 to +275ms |
|  | 6 | [55.3 3.8 31.4] | Inferior Frontal Gyrus (Brodmann Area 6) | +125 to +275ms |
|  | 7 | [55.3 26.0 14.0] | Inferior Frontal Gyrus (Brodmann Area 9) | +125 to +325ms |
|  | 8 | [55.3 -67.5 -41.3] | Cerebellar Posterior Lobe | +175 to +325ms |
|  | 9 | [18.2 52.9 38.6] | Superior Frontal Gyrus (Brodmann Area 9) | +225 to +325ms |
|  | 10 | [15.0 62.4 7.7] | Superior Frontal Gyrus (Brodmann Area 10) | +375 to +425ms |
|  | 11 | [55.3 -52.5 -43.7] | Cerebellar Tonsil | +375 to +425ms |
|  | 12 | [45.8 -66.7 45.7] | Inferior Parietal Lobule (Brodmann Area 39) | +375 to +425ms |
|  | 13 | [63.2 -12.1 -16.0] | Middle Temporal Gyrus (Brodmann Area 21) | +425ms |

**Supplementary Table 8: Peak voxels with significant high-gamma-band activity differences with respect to pitch perturbation onset**

| **Hemisphere** | **Peak** | **MNI Coordinates** | **Anatomical Labels** | **Time with respect to pitch perturbation onset** |
| --- | --- | --- | --- | --- |
| Left | 1 | [-32.0 29.0 7.0] | Inferior Frontal Gyrus (Brodmann Area 45) | +25 to +125ms |
|  | 2 | [10/9 35.1 -26.5] | Rectal Gyrus (Brodmann Area 11) | +25 to +425ms |
|  | 3 | [-38.1 -42.0 -54.0] | Cerebellar Tonsil | +25 to +425ms |
|  | 4 | [-31.1 -81.9 38.1] | Angular Gyrus (Brodmann Area 39) | +175 to +275ms |
|  | 5 | [-48.0 -20.7 -34.3] | Inferior Temporal Gyrus (Brodmann Area 20) | +225 to +325ms |
| Right | 1 | [52.3 37.2 17.6] | Middle Frontal Gyrus (Brodmann Area 46) | +25 to +225ms |
|  | 2 | [14.6 29.3 -25.0] | Orbital Gyrus (Brodmann Area 47) | +125 to +425ms |
|  | 3 | [56.0 -27.5 -24.9] | Inferior Temporal Gyrus (Brodmann Area 20) | +125 to +425ms |
|  | 4 | [48.0 45.0 23.0] | Middle Frontal Gyrus (Brodmann Area 46) | +125 to +225ms |
|  | 5 | [56.3 -74.0 -33.0] | Cerebellar Posterior Lobe | +175 to +425ms |
|  | 6 | [8.0 -91.0 31.0] | Cuneus (Brodmann Area 19) | +175 to +275ms |
|  | 7 | [46.0 -84.3 -16.2] | Inferior Occipital Gyrus (Brodmann Area 18) | +225 to +425ms |
|  | 8 | [24.0 33.3 54.6] | Superior Frontal Gyrus (Brodmann Area 8) | +325ms |

**Abbreviations used: fMRI = Functional Magnetic Resonance Imaging, PET = Positron Emission Tomography, EEG = Electroencephalography, L = left, R = right, M1 = primary motor cortex, S1 = primary somatosensory cortex, ACC = anterior cingulate cortex, SMA = supplementary motor area, dPMC = dorsal premotor cortex, SFG = superior frontal gyrus, IFG = inferior frontal gyrus, MFG = middle frontal gyrus, STG = superior temporal gyrus, MTG = middle temporal gyrus, A1 = primary auditory cortex, SII = secondary somatosensory cortex, SMG = supramarginal gyrus, PAG = periaqueductal grey, MCC = middle cingulate cortex, ITG = inferior temporal gyrus.**
